# Tape-assisted fabrication method for constructing PDMS membrane-containing culture devices with cyclic radial stretching stimulation

**DOI:** 10.1098/rsos.240284

**Published:** 2024-08-14

**Authors:** Yun-Chen Wu, Jing-Yi Yang, Chia-Hsien Hsu

**Affiliations:** ^1^ Institute of Biomedical Engineering and Nanomedicine, National Health Research Institutes, Zhunan, Miaoli 35053, Taiwan; ^2^ Institute of Nanoengineering and Microsystems, National Tsing Hua University, Hsinchu 30013, Taiwan; ^3^ Doctoral Program in Tissue Engineering and Regenerative Medicine, National Chung Hsing University, Taichung 40227, Taiwan

**Keywords:** microfabrication, PDMS membrane, microphysiological systems, YAP/TAZ

## Abstract

Advanced *in vitro* culture systems have emerged as alternatives to animal testing and traditional cell culture methods in biomedical research. Polydimethylsiloxane (PDMS) is frequently used in creating sophisticated culture devices owing to its elastomeric properties, which allow mechanical stretching to simulate physiological movements in cell experiments. We introduce a straightforward method that uses three types of commercial tape—generic, magic and masking—to fabricate PDMS membranes with microscale thicknesses (47.2 µm for generic, 58.1 µm for magic and 89.37 µm for masking) in these devices. These membranes are shaped as the bases of culture wells and can perform cyclic radial movements controlled via a vacuum system. In experiments with A549 cells under three mechanical stimulation conditions, we analysed transcriptional regulators responsive to external mechanical stimuli. Results indicated increased nuclear yes-associated protein (YAP) and transcriptional coactivator with PDZ-binding motif (TAZ) activity in both confluent and densely packed cells under cyclically mechanical strains (Pearson’s coefficient (PC) of 0.59 in confluent and 0.24 in dense cells) compared with static (PC = 0.47 in confluent and 0.13 in dense) and stretched conditions (PC = 0.55 in confluent and 0.20 in dense). This technique offers laboratories without microfabrication capabilities a viable option for exploring cellular behaviour under dynamic mechanical stimulation using PDMS membrane-equipped devices.

## 1. Introduction

*In vitro* models have served as fundamental platforms for the advancement of biomedical research and preclinical studies. Cells and microtissues cultured *in vitro* provide insights into cell behaviours and biological phenomena under controlled conditions, offering simplicity and ease of manipulation for scientists when compared with *in vivo* studies such as those involving animals. However, these models are constrained by artificial environments that do not replicate native growth conditions [1]. Traditional cell culture techniques, such as the use of Petri dishes and culture well plates with rigid, two-dimensional bottoms, are limited in their ability to mimic natural environmental cues [2,3]. In the past two decades, on-chip devices or advanced microphysiological systems have been developed to recapitulate the complex microenvironment and microphysiology of living cells and tissues cultured on a microscale [4–6].

In addition to advancing conventional cell culture plasticware, there is a pressing need to develop advanced microphysiological systems to reduce or even replace the use of experimental animals [7]. A key concern is the treatment and welfare of animals, which has increasingly attracted societal scrutiny [8]. Another issue is that animal testing is often lengthy, costly and can yield misleading results [9]. A critical concern is the limited ability of laboratory animals to accurately predict human drug responses, impacting the safety and effectiveness of clinical trials. Hence, the reliance on preclinical animal models has been re-evaluated [10]. Microphysiological systems, or organ chip systems, are now considered promising alternative methods that can support therapeutic development and minimize animal use [7,11].

In biomedical research, advanced cell culture systems serve as a versatile toolkit, enabling scientists to design and enhance their experiments [4]. Polydimethylsiloxane (PDMS), favoured for its ease of moulding into various structural shapes and its properties such as transparency and biocompatibility, is extensively used as the foundational material in constructing advanced microphysiological systems. These systems provide critical anatomical and physiological cues for cell experiments [12,13]. For instance, Grosberg *et al*. developed a muscular thin film on-chip system by using a spin-coated PDMS layer patterned as substrates for muscle cell culture, incorporating contractility assays [14]. Additionally, varying the cross-linker concentrations in the PDMS curing process allows for adjustments in the stiffness of PDMS-based culture devices. This variation in stiffness can influence cell mechanotransduction, enabling the study of the pathophysiological processes in vascular diseases [15].

The most well-known PDMS membrane-containing device is the pioneering lung-on-chip system, which uses a vacuum to apply tension on an elastic PDMS membrane, creating breathing-like movements for the cultured cells [16]. Stucki *et al*. developed an alveolus-on-chip model featuring a PDMS micro-diaphragm membrane attached to the base, designed to contract and simulate cyclic breathing strains on the cultured cells [17,18]. Beyond lung models, PDMS-based stretching devices are also employed to study the effects of mechanical forces on the biology of heart muscle cells [19,20]. Exploring further, Man *et al*. investigated how cells respond when cultured on PDMS membranes of varying geometries that can be mechanically stretched in uniaxial, circumferential and radial forms [21].

However, widespread adoption of these platforms in the scientific community is limited owing to the specialized equipment required, including a yellow cleanroom and a soft lithography aligner [22], a spin coater for creating micro-scale membranes [23,24] and an oxygen plasma system for material bonding [25]. This article presents a simpler alternative method that only requires a desktop computer numerical control (CNC) machine to create the base tooling for PDMS moulding. Using common adhesive tapes, this technique achieves a one-piece moulding process that circumvents the need for the standard oxygen plasma treatment for PDMS bonding [26]. When combined with a vacuum setting, the membrane can perform radial motions that mimic physiological activities, such as breathing movements for cell culture.

To demonstrate the utility of the device, we conducted experiments using A549 human lung epithelial cells, a cell line well suited for studying biological responses in lung chip devices [27–29]. We investigated cellular expressions of yes-associated protein (YAP) and transcriptional coactivator with PDZ-binding motif (TAZ), both of which play roles in mechanotransduction [30–32]. YAP/TAZ proteins transition between the cytoplasm and nucleus, influenced by extracellular mechanical signals or cytoskeletal structures, affecting nuclear YAP/TAZ activity. Within the nucleus, YAP/TAZ act as transcription factors, partnering with other proteins to regulate gene transcription [33]. In our system, cyclic stretching serves as an external mechanical cue to simulate physical respiration. Immunofluorescence imaging was used to analyse nuclear YAP/TAZ activity under static, stretched and dynamic mechanical conditions. Results indicated that nuclear YAP/TAZ activity was higher in stretched cells when compared with static ones. To simulate cyclic movements such as breathing, we assessed the dynamic group, where the nuclear colocalization of YAP/TAZ was the most pronounced among the three mechanical states.

## 2. Material and methods

### 2.1. Fabrication of one-piece made construct

**Figure 1.**
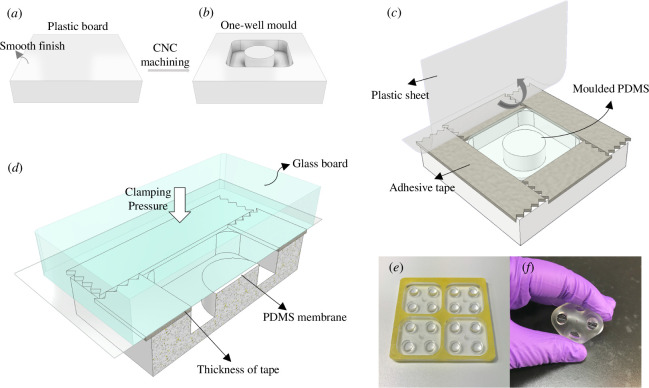
Fabrication of the one-piece PDMS construct. (*a*–*d*) Schematic illustration of the fabrication process. (*a*) Plastic board with a smooth finish is used as the raw material for the CNC machining process. (*b*) Plastic mould is created with a smooth finish on the surface for the central post, which is level with the surrounding walls, to mould the bottom of cell culture wells. (*c*) Fabrication of the one-piece moulded PDMS construct involves placing adhesive tapes around the mould, pouring PDMS into the mould and covering it with a plastic sheet for clamp moulding. (*d*) The cross-sectional view shows the thickness of the PDMS membrane (not to scale), with a glass board placed on top of the plastic sheet to squeeze out excess PDMS along the flat surface under clamping pressure. (*e*) The mould designed for producing 4-well PDMS constructs. (*f*) The final product is a one-piece PDMS construct featuring four culture wells with bottoms made of elastic membranes.

To fabricate one-piece PDMS constructs with an integrated elastic membrane for culture devices, we prepared a plastic mould using CNC machining (Roland MDX-40) for rapid prototyping. A poly(methyl methacrylate) (PMMA) board served as the raw material for the mould. Figure 1*a,b* depicts the mould schematically, highlighting the smooth-finished surface of the culture well, untouched by CNC machining. Figure 1*e* shows the mould used for this study, designed for four 4-well constructs. Each cell culture well measures 7 mm in diameter and 3 mm in depth. To create microscale membranes, adhesive tapes were applied around the moulding site to create a micrometre-thick gap. Uncured PDMS was then poured into this gap, forming the membrane that merged seamlessly into the wall of a culture well (figure 1*c,d*). The moulding process began by mixing PDMS pre-polymer (Dow Corning Sylgard 184) with its curing agent at a 10 : 1 ratio. This mixture was then degassed in a vacuum desiccator to eliminate air bubbles. A plastic sheet was carefully laid over the uncured PDMS to prevent further bubble formation. A thick, flat glass plate was then placed on top of the plastic sheet for clamp moulding, applying direct clamping force to squeeze out excess PDMS from the moulding site (figure 1*d*) [34]. The moulds were then cured in a 65°C oven for at least 3 h. After curing, the PDMS constructs were carefully peeled from the mould. Figure 1*f* illustrates a 4-well construct with elastic membranes formed at the bottoms of the culture wells.

### 2.2. Measurements of tape-thick PDMS membrane

**Figure 2.**
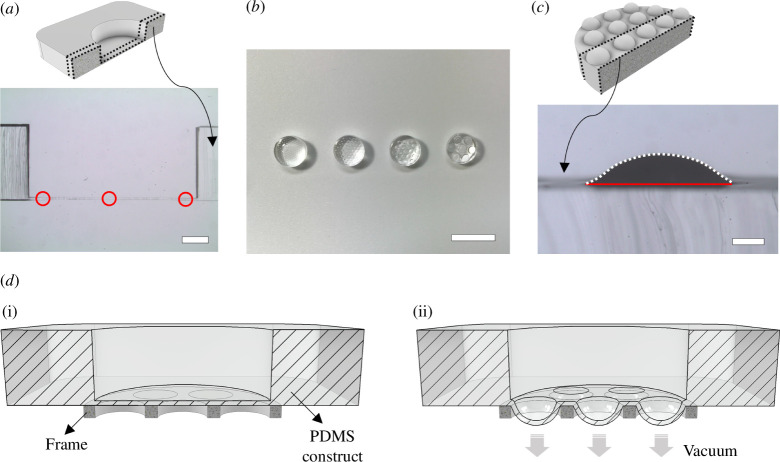
Characterization of the tape-thick PDMS membrane. (*a*) The sectional view of a PDMS construct well, with membrane thickness measured at three positions (indicated by red circles) and averaged (scale bar = 1 mm). A schematic view of the cut slice for measurement is shown above. (*b*) Moulded PDMS replicas arranged from left to right, created with hole diameters of 250, 500, 1000 and 2000 µm in the frames (scale bar = 10 mm). (*c*) Strain measurements of replicas: the white dotted line represents the length of the strained membrane, and the red bottom line indicates the original length of the flat membrane without straining (scale bar = 200 µm). A schematic view of the cut slice for measurement is shown above. (*d*) The schematic of alveolar-like stretching formation: (*d*(i)) shows a sectional view of the membrane in a static state (no vacuum applied); (*d*(ii)) depicts the membrane in a stretched state (with vacuum applied).

To illustrate the method of incorporating tape-thick PDMS membranes into the culture device, three types of tape were used: a generic brand of transparent tape (generic), 3M Scotch Magic 810 (magic) and 3M Scotch Masking 244 (masking). The cured PDMS constructs were transversely cut using a razor blade to display the thicknesses of the membranes. These membrane thicknesses shown by the cut slices were measured using a stereomicroscope (Nikon SMZ1500) and image analysis software (QCapture Pro). For each type of tape, five sample constructs were analysed. Measurements are taken at three locations on each membrane (left side, right side and centre, as shown in figure 2*a*), and the results are averaged.

### 2.3. Characterization of membrane

To understand the stretching effect on the PDMS membrane, rigid plastic frames, comprising multiple circular holes, were directly placed at the membrane’s bottom to generate a radial stretch under the vacuum applied (figure 2*d*). There are four groups of hole diameters, namely 250, 500, 1000 and 2000 µm of the frames. Furthermore, four different vacuum strengths, −20, −40, −60 and −80 kPa were applied under the membranes. To characterize the results of vacuum strengths exerted on the membranes with radial stretches, the PDMS replica moulding process [35,36] was used to acquire structural formation (figure 2*b*). The strains of each radial stretch were measured by transversely cutting moulded replicas using a razor blade from the top to the bottom of the replicas. The strains were calculated: $\frac{\Delta L\;\left(stained\;line-original\;line\right)}{L\;\left(original\;line\right)}$ using ImageJ to acquire the length of the strained lines and the original line for the calculations (figure 2*c*). Curvature values of the strained lines were calculated using the mean value of all the curvature points measured by ImageJ with Kappa. Five measurements from each group were considered and analysed.

### 2.4. Setting for cell experiments

**Figure 3.**
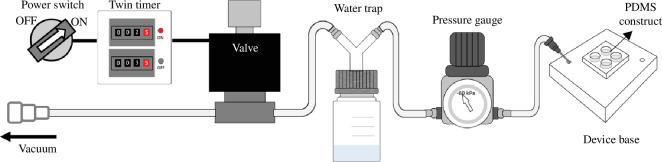
The assembled setting for cell experiments.

Figure 3 schematically illustrates the cell culture set-up used in this study. An electromagnetic valve (CKD AG41-03-1), controlled by a twin timer (Airtac GVR300 or SMC IRV20), was electronically assembled to regulate the timing of vacuum pressure applied to the elastic membrane. This set-up allows the membrane to perform dynamic motions mimicking cyclic breathing movements (refer to electronic supplementary material, video S1). We set the cycle duration to 5 s to emulate a typical human respiratory rate of 12 breaths per minute. A water trap, created by modifying the lid of a lab glass bottle with a two-way connector glued on, captures moisture from the culture incubator and buffers airflow when the vacuum system operates. This prevents water accumulation in the connecting tubes and devices that could impair their function. To control the strength of the vacuum pressure, a negative pressure gauge was connected at the base of the device with a tube ending in a needle to provide the vacuum. Positioned above the water trap on top of the incubator, the gauge’s placement prevents moisture accumulation inside it, ensuring functionality after multiple sets of cell experiments.

The device, covered by a transparent plastic lid (not shown), was placed in a 37°C incubator with 5% carbon dioxide. The base of the device, designed to hold PDMS constructs, features a hollow chamber for applied vacuum. The top part of the base includes structures for positioning the PDMS constructs where the membrane is supported on a multi-circular frame and includes two tiny holes for the inlets of vacuum and atmosphere.

### 2.5. Cell maintenance

A549 human epithelial cells (CCL-185) were cultured in a 37°C incubator with 5% CO_2_. The cells were grown and expanded in 100 mm dishes using Dulbecco’s modified Eagle medium (Gibco 12100046) with 10% fetal bovine serum (Millipore TMS-013-BKR) and 1% penicillin-streptomycin (Gibco 15140122). The culture medium was refreshed and cells were passaged twice weekly for maintenance. For this study, A549 cell passages from 16 to 25 were used.

### 2.6. Cell culture on PDMS constructs

The day before cell seeding, PDMS membranes were coated with human plasma fibronectin (Merck FC010) at a concentration of 20 µg ml^−1^ in phosphate-buffered saline (PBS). The membranes were submerged in the fibronectin solution at 4°C overnight for physical adsorption [37,38]. Prior to seeding, the fibronectin-coated membranes were washed twice with PBS. A549 cells were then seeded onto the PDMS membranes at varying densities—2.5 × 10^4^ cells for confluent coverage and 10 × 10^4^ cells for denser coverage per culture well. The cells were maintained in the incubator for 2 days before any further experimental procedures were conducted.

### 2.7. Mechanical experiments on cells

After a 2-day cell culture on the elastic membrane, the experiments using the assembled setting (figure 3) were conducted to understand cellular behaviour in different mechanical states: static, stretched and dynamic. To test the applicability of our devices, the static state was without any mechanical handling; the stretched stage corresponded to the membrane stretched on a 2000 µm diameter multi-circular frame under vacuum applied at −60 kPa for 3 h. The dynamic state corresponded to the membrane on a multi-circular frame with a diameter of 2000 µm, which moved dynamically owing to the controlling effect of the twin timer set to be on for 2 s and off for 3 s, thus creating a cycle of 5 s per cycle (0.2 Hz) over a duration of 3 h.

### 2.8. Immunofluorescence

A549 cells were fixed on the membranes inside the wells using 4% formaldehyde for 12 min and followed by washing the cells with 1 × PBS three times. Then, the cells were permeabilized using 0.2% Triton X100 (Sigma 9002-93-1) in PBS for 10 min and a blocking procedure using 5% bovine serum albumin (BSA) in 1 × PBS with 0.1% triton X100 (PBST) for at least 30 min at room temperature. The immunostaining was accomplished by incubating cells with primary antibodies at 4°C overnight. After three washes with PBST, the secondary antibodies were incubated for at least 2 h at room temperature, followed by three additional washes with PBST. Finally, cells were stained with Hoechst 33 342 (Abcam ab228551) (5 mM in PBS) for 25 min followed by two washes with PBS before microscopy. The antibodies used in this study were anti-YAP/TAZ (Santa Cruz sc101199) 1 : 200 and anti-zonula occludens-1 (ZO1) (Abcam ab221547) 1 : 200 for primary antibodies, and secondary antibodies conjugated to Alexa488 (Abcam ab150113) and Alexa647 (Abcam ab150075). All antibodies were diluted in 2% BSA in PBST for immunostaining. Staining without primary antibodies was used as the negative control. A Leica TCS SP5 II confocal microscope was used to take the immunofluorescent images. Cells of the part on the circular hole of the frame were observed.

### 2.9. Image analyses

The fluorescence intensity of the nuclear-to-cytoplasmic ratio [21,39] was analysed using ImageJ. The Hoechst staining sites were selected for calculating the mean intensity value of nuclear YAP/TAZ staining and non-nuclear sites for cytoplasmic YAP/TAZ staining. The co-localization analysis to quantify subcellular co-localization of nuclear and YAP/TAZ stains was performed using ImageJ software with a plug-in app JACoP BIOP version to generate the scatter plot and calculate the Pearson’s coefficient of each set of fluorescent images [40].

### 2.10. Statistical analyses

GraphPad Prism and Microsoft Excel were used for statistical analyses and plotting. One-way ANOVA with Tukey’s multiple comparisons was used. Specifically, *p*-values of *p < 0.05, **p < 0.005, ***p < 0.001 and ****p < 0.0001 were considered as statistically significant.

## 3. Results

### 3.1. Characterization of tape-thick PDMS membrane

**Figure 4.**
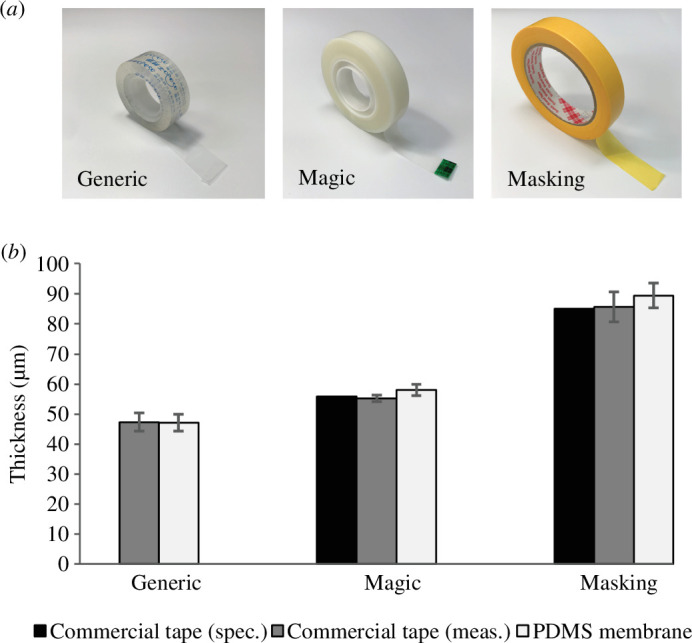
Comparison of thicknesses for commercial tapes and PDMS membranes produced using a tape-assisted method. (*a*) Images of commercial tapes arranged from left to right: a generic brand of transparent tape (generic), 3M Scotch Magic 810 (magic) and 3M Scotch Masking 244 (masking). (*b*) Thickness comparison of commercial tapes according to specifications (in black columns), measurements obtained using microscopic analysis software (in dark grey columns) and PDMS membranes fabricated with different tapes (*n* = 5; mean with standard deviation displayed).

The moulded constructs for cell culture demonstrated robust integration between the membrane and main body, achieved through the one-piece PDMS clamp moulding process previously described. This microscale membrane effectively satisfies the elastic demands under mechanical force, as shown in figure 1*f*. To assess the feasibility of using this method to produce microscale PDMS membranes, three commercial tapes of varying micrometre thicknesses are employed in the fabrication process, as depicted in figure 4*a*. The thickness measurements from the specification data of these tapes included 55.88 µm for magic tape, commonly used in our lab for cell culture, and 85 µm for masking tape (indicated by the black columns in figure 4*b*). However, no specification data could be obtained for the generic tape, typically used in office settings. A microscopic analysis using QCapture Pro software confirms similar thicknesses to those specified, as shown in the dark grey columns in figure 4*b*. Following the tape-assisted fabrication method for creating PDMS membranes, the generic tape resulted in the thinnest membrane, approximately 47.2 µm. Conversely, the membranes fabricated with magic tape and masking tape were slightly thicker than their commercial counterparts, measuring 58.1 and 89.37 µm, respectively (represented by the light grey columns in figure 4*b*).

**Figure 5.**
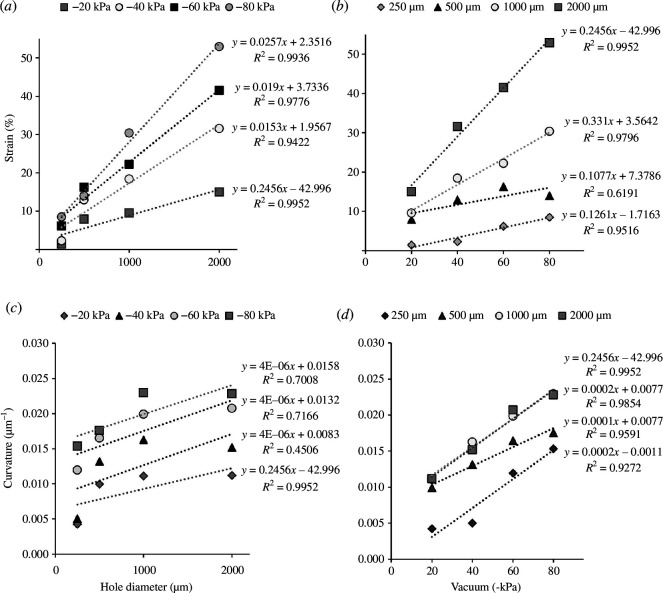
PDMS membrane stains and curvature. (*a*) Plot of the linear regression trendline on the hole diameter of the frames versus strain (*n* = 5). (*b*) Plot of the linear regression trendline on the vacuum strength versus the strain (*n* = 5). (*c*) Plot of the linear regression trendline on the hole diameter of the frames versus curvature (*n* = 5). (*d*) Plot of the linear regression trendline on the vacuum strength versus curvature (*n* = 5).

To visualize the radial stretching effect, PDMS membrane constructs, created using magic tape, were subjected to replica moulding under mechanical strain. The strains observed in the moulded replicas were then compared. Linear regression trendlines were plotted using the measured strain data to analyse the relationships between the various parameters. Under stretching forces, the strains increased linearly in relation to the size of the multi-circular frame at the bottom, yielding *R* ^2^ values ranging from 0.94 to 0.99 across four vacuum strengths (figure 5*a*). Conversely, the predictability of vacuum strength was assessed using the same frame size, revealing that the strains in the membranes increased with the pressure applied, establishing a linear correlation between strain and pressure as depicted in figure 5*b*. In terms of membrane curvature, only the samples under a lower vacuum strength of −20 kPa demonstrated a high linear correlation between curvature and hole diameter, with an *R* ^2^ value of nearly 1 (figure 5*c*). When comparing this relationship, a higher linear correlation between curvature and vacuum strength was observed, with *R* ^2^ values exceeding 0.92 under four different vacuum strengths (figure 5*d*).

### 3.2. Influence of cell density on YAP/TAZ translocation

**Figure 6.**
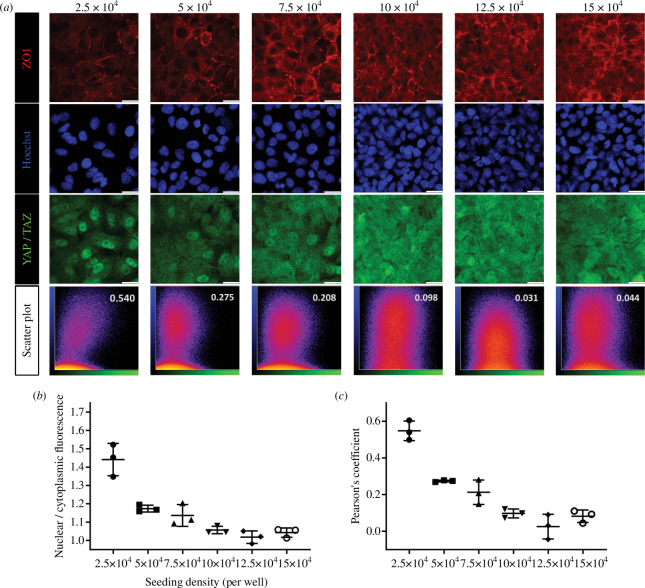
Influence of seeding density on YAP/TAZ translocation. (*a*) Various A549 seeding densities displayed from left to right: 2.5 × 10^4^, 5 ×10^4^, 7.5 ×10^4^, 10 ×10^4^, 12.5 ×10^4^ and 15 × 10^4^ cells per well in a PDMS construct. After 2 days of culture, cells were fixed and immunostained with anti-ZO1 (red), anti-YAP/TAZ (green) and Hoechst stain. Representative images from each density are shown with a white scale bar of 20 µm at the bottom right of each image. Scatter plots for each density were analysed using ImageJ, with Pearson’s coefficients displayed at the top right of the plots. (*b*) Comparison of nuclear to cytoplasmic fluorescence intensity across different seeding densities. (*c*) Comparison of Pearson’s coefficients across seeding densities, with three sets of fluorescent images analysed for each group (mean with s.d.).

Before assessing the mechanical action of elastic PDMS membranes for cell culture, the optimal seeding density was established. YAP/TAZ, termed as transcriptional regulators, enable cells to respond to mechanical cues within their microenvironments [31,41]. Previous studies indicated that seeding density significantly affects the percentage of nuclear YAP/TAZ staining. High-density seeding resulted in transcriptional inactivation of YAP/TAZ, with immunostaining primarily observed in the cytoplasm. Conversely, sparsely seeded and confluent cells exhibited elevated nuclear YAP/TAZ levels, indicating active transcription [42,43]. To determine the appropriate cell density for this study, A549 cells were cultured at six different seeding densities (2.5 × 10^4^, 5 × 10^4^, 7.5 × 10^4^, 10 × 10^4^, 12.5 × 10^4^ and 15 × 10^4^) for 2 days on a fibronectin-coated PDMS membrane in a culture well of a fabricated construct (figure 1*f*). The immunofluorescent results across these densities are displayed in figure 6. Anti-ZO1, a marker for tight junction proteins in epithelial cells, was used for immunostaining to delineate cellular borders [44,45]. To quantify nuclear YAP/TAZ activity, the co-localization of nuclear (Hoechst stain) and YAP/TAZ stains was analysed using Pearson’s correlation coefficient [40,46]. Scatter plots of this analysis are presented in the bottom row of figure 6*a*, and comparisons of nuclear-to-cytoplasmic fluorescence intensity are shown in figure 6*b*. Cellular borders and nuclear stains appeared denser from partially sparse or confluent to dense seeding densities up to around 10 × 10^4^ per culture well, where the immunostaining of junctional protein ZO1 and nuclei reached nearly the same confluence as seen in larger seeding densities of 12.5 × 10^4^ and 15 × 10^4^. At the lowest seeding density in this study, cells demonstrated clear nuclear YAP/TAZ localization with bright fluorescent sites coinciding with nuclear stains, resulting in the highest Pearson’s coefficient among the six densities. At densities higher than 10 × 10^4^, YAP/TAZ staining was less distinct at nuclear sites when compared with lower densities, with fluorescence spreading to both nuclear and cytoplasmic areas, and some nuclear areas showing reduced or absent YAP/TAZ staining. Analysis of nuclear-to-cytoplasmic fluorescence intensity and Pearson’s coefficients across six seeding densities indicates that the values increased up to a seeding density of 10 × 10^4^ per well.

### 3.3. Influence of mechanical states on nuclear YAP/TAZ activity

**Figure 7.**
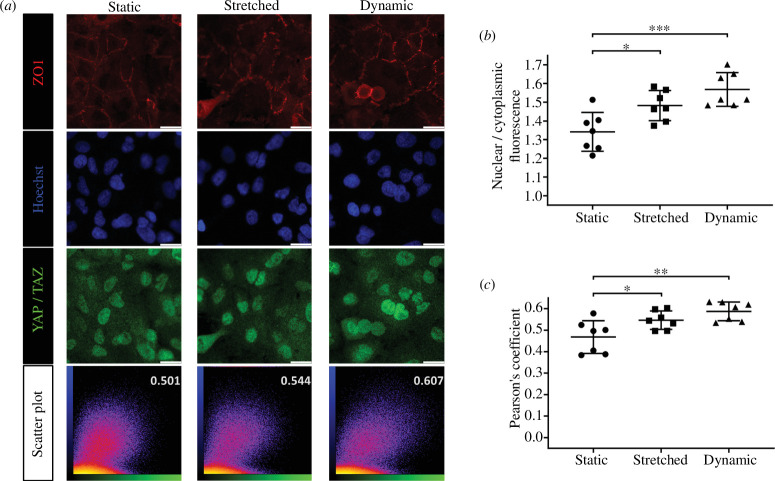
Impact of mechanical states on confluent cells (2.5 × 10^4^ cell seeding density for 2-day culture per well). (*a*) Representative immunofluorescent images and scatter plots of confluent cells subjected to static, stretched and dynamic mechanical conditions. (*b*) Comparison of nuclear to cytoplasmic fluorescence intensity ratios across different mechanical states. (*c*) Comparison of PCs for the three mechanical states, with analyses performed on seven sets of imaging data for each condition (mean with s.d.; *p < 0.05, **p < 0.005, ***p < 0.001).

Based on previous experimental results, a seeding density of 2.5 × 10^4^ cells was used for confluent cultures, and 10 × 10^4^ for dense cultures to further study mechanical effects. Figure 7 presents representative immunostaining images of confluent cells under three different mechanical states. The static state involved no manipulation, with cells cultured for 2 days in an incubator before fixation. Conversely, the stretched state involved the PDMS membrane being subjected to continuous mechanical stretching for 3 h after a 2-day culture period. The dynamic state involved cyclic mechanical strain applied for 3 h on the second day of culture, mimicking the physiological radial stretching of the cultured membrane. YAP/TAZ staining primarily coincided with nuclear stains in confluent cells (figure 7*a*). When quantifying using the ratio of nuclear to cytoplasmic fluorescence intensity and PC analysis, differences among the three mechanical states, not visually discernible, are depicted in figure 7*b*,*c*. Cells under cyclic mechanical stretch (dynamic) exhibited the highest PC, indicating the most robust nuclear YAP/TAZ activity under dynamic conditions compared with stretched and static states. Conversely, the lowest nuclear co-localization of YAP/TAZ was observed in cells under the static state.

**Figure 8.**
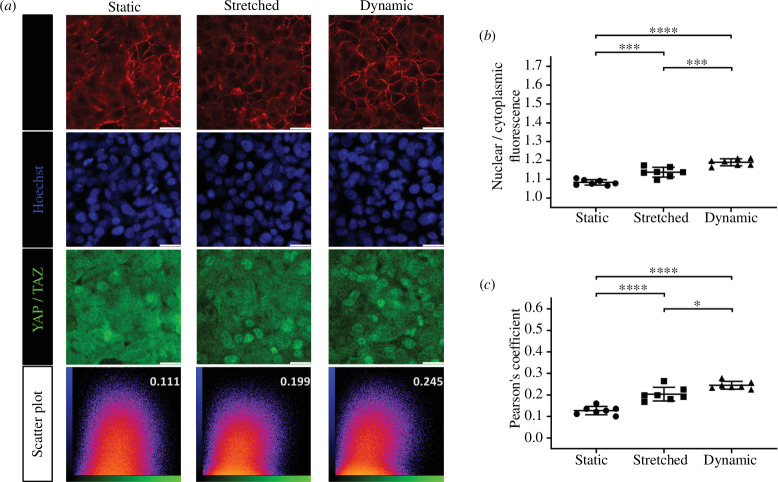
Influence of mechanical states on dense cells (10 × 10^4^ cells seeding density for 2-day culture per well). (*a*) Representative immunofluorescent images and scatter plots of dense cells in the static, stretched and dynamic mechanical states. (*b*) Comparison of the nuclear/cytoplasmic fluorescence intensity of different seeding densities. (*c*) Comparison of PCs for the three mechanical states, with analyses performed on seven sets of imaging data for each condition (mean with s.d.; *p < 0.05, ***p < 0.001, ****p < 0.0001).

When comparing three mechanical states in dense cells, nuclear YAP/TAZ localization was clearly visible in the immunostaining images of cells in both the mechanically stretched and dynamic states. However, it was not clear in the static state (figure 8*a*). The differences were evident when analysing the ratio of nuclear to cytoplasmic fluorescence intensity and PCs (figure 8*b,c*). Similar to confluent cells, dense cells exhibited the highest nuclear YAP/TAZ localization under dynamic conditions when compared with static and stretched states, with cells in the static state showing the lowest nuclear co-localization of YAP/TAZ.

## 4. Discussion

Over the past two decades, the development of *in vitro* cell culture platforms that mimic human physiology has thrived in the study of various disease models and the exploration of effective therapeutics [47]. The design and fabrication decisions of these devices are critical and closely tied to their intended applications [4]. The advent of microsystems technology, largely propelled by the established techniques of soft lithography, has led to numerous innovations in microfluidic devices and cell culture models. These techniques allow for diverse device configurations, such as microfluidic channels and multiple culture chambers, enabling the simulation of cell-to-cell interactions within biological environments on a microscale. PDMS, known for its optical properties, low toxicity and biocompatibility, is one of the most commonly used materials in the fabrication of lithographically patterned devices [48]. This popularity dates back to early works in microfluidics. We have leveraged the advantageous properties of PDMS, including its excellent elasticity, to fabricate cell culture devices that simulate cyclic physiological motion.

Adhesive tape has facilitated scientific breakthroughs in research like X-ray generation through tape peeling [49] and the preparation of thin graphene films using the Scotch tape method [50], which ushered in a new era of graphene research [51]. Inspired by these innovations, we employed tape in the fabrication process of PDMS devices to create cell culture devices with integrated microscale-thickness PDMS membranes, where the membrane thickness is dictated by the tape used. Conventionally, PDMS membrane thickness is adjusted by varying the spin coating parameters of the PDMS pre-polymer [23,35]. Traditional PDMS membrane-containing microfluidic devices also require oxygen plasma treatment to bond different structural components, a procedure where variations in operational processes can affect adhesive quality or lead to bonding failures [52,53]. Our method circumvents the need for oxygen plasma bonding by curing the PDMS membrane and device body as a single-piece construct, which enhances the structural integrity and robustness of the resulting constructs [54].

Rapid prototyping is a swift and efficient method for fabricating objects directly from computer-aided designs, commonly used to create moulds for PDMS-based cell culture devices [55,56]. Current commercial rapid prototyping machines, both additive (e.g. three-dimensional printing for material deposition) and subtractive (e.g. CNC machining and laser cutting for material removal) [57,58], often struggle to achieve the smooth surfaces that are typical of plastic injection moulding used in industrial mass production. In our study, CNC machining, a subtractive method where solid material is carved into designed shapes, was employed to create the base tooling for the PDMS constructs. However, this process introduced surface roughness owing to the drilling action. To counteract this, we used a plastic board with a pre-existing smooth finish for the bottom of the culture wells, which we intentionally did not subject to CNC machining (figure 1). The integration of adhesive tapes in the PDMS moulding process enabled the formation of flat, transparent membranes with excellent optical properties, suitable for microscopy and biological assays.

While some protocols use surface treatments to achieve smooth finishes on moulds [56,59,60], our process minimizes both processing time and chemical use in creating master moulds. After the CNC machining process, we intentionally did not machine the areas designated for the PDMS membrane, ensuring these surfaces remained smooth for the bottoms of culture wells. In addition to streamlining device fabrication, we designed a simple, easy-to-assemble system to control the vacuum (figure 3). This system uses only a modified laboratory bottle as a water trap and inexpensive, readily available components from hardware stores. This set-up provides a practical platform for researchers in standard biology laboratories, enabling them to study cellular behaviour under dynamic mechanical stimulation without the need for specialized microfabrication facilities.

A study analysed the effects of different types of mechanical stretching—uniaxial, circumferential and radial—on cell behaviours. Unlike cells that elongate perpendicularly to the direction of uniaxial and circumferential stretches, cells under radial mechanical stretch tend to orient randomly, a configuration suggestive of better epithelium maintenance [21]. Similarly, in our system, A549 cells subjected to radial mechanical strains did not elongate in a specific direction but oriented randomly. To fully understand cell morphology under stretching and the impact of actual stimulation at various points within the radially stretched area, live cell imaging is essential. However, our current culture device, equipped with a vacuum system, is technically limited in capturing the elongation ratio of cells during stretching. Future enhancements to our system should include modifications to accommodate a microscopic set-up for live cell imaging.

YAP/TAZ, identified as transcriptional regulators in the mechanotransduction of mechanical signals from their microenvironments, are influenced by the engineered mechanical properties of *in vitro* models. Adjusting matrix stiffness [30,61,62], patterning substrate topological features [63,64] and applying mechanical stretching to cells [39,43] have all been shown to regulate cellular YAP/TAZ activity. Studies have shown that both static [43] and cyclic [39] stretching forces increase YAP/TAZ nuclear localization. In our study, dynamic mechanical stimulation, simulating cyclic physiological motion, resulted in the highest nuclear YAP/TAZ activity in both confluent and dense cells compared with static and stretched states.

The stretching experiments conducted in this study subjected A549 cells within 2000 µm circular frames to a −60 kPa stretching force, testing the feasibility of our device fabrication approach with our in-house vacuum system. For a more accurate physiological emulation of alveolar movements, future studies could use smaller stretching frames (e.g. 200 µm diameter) and vary vacuum strengths to simulate both normal and aberrant mechanical states. Additionally, incorporating non-cancerous cell lines or primary lung epithelial cells [65] could provide a more intricate physiological mimicry, enhancing our understanding of pathological effects in lung diseases.

## 5. Conclusion

In this study, we introduce an alternative method for fabricating PDMS culture devices with elastic membranes, eliminating the need for conventional microfabrication facilities. We use commercial tapes to create thin PDMS membranes with microscale thicknesses (generic 47.2 µm, magic 58.1 µm and masking 89.37 µm), enabling cyclic motion to simulate physiological radial movements like respiration in cultured cells. Using these devices, we conducted co-localization analyses of nuclear YAP/TAZ expressions under three different mechanical states. The results highlighted increased nuclear YAP/TAZ activity in cells subjected to dynamic mechanical strains, with Pearson coefficients (PC) of 0.59 in confluent cells and 0.24 in dense cells, compared with static (PC = 0.47 in confluent and PC = 0.13 in dense) and stretched states (PC = 0.55 in confluent and PC = 0.20 in dense). This fabrication method could also be adapted to develop *in vitro* culture models that dynamically stimulate PDMS membranes for enhanced physiological mimicry.

## Data Availability

The datasets supporting the article were uploaded as part of the electronic supplementary material [66,67].
